# Anemia can predict the prognosis of colorectal cancer in the pre-operative stage: a retrospective analysis

**DOI:** 10.1186/s12957-021-02452-7

**Published:** 2021-12-08

**Authors:** Rotem Gvirtzman, Dan Meir Livovsky, Esther Tahover, Eran Goldin, Benjamin Koslowsky

**Affiliations:** 1 Faculty of Medicine, Hebrew University of Jerusalem, Digestive Diseases Institute, Shaare-Zedek Medical Center, Jerusalem, Israel; 2 Faculty of Medicine, Hebrew University of Jerusalem, Oncology Department, Shaare-Zedek Medical Center, Jerusalem, Israel

**Keywords:** Advanced cancer, Anemia, Colorectal cancer, Gastrointestinal malignancy, Iron deficiency anemia, Screening colonoscopy

## Abstract

**Background:**

Early detection of colorectal cancer (CRC) improves prognosis, yet many CRCs are diagnosed following symptoms. The aim of this study was to determine which CRC-related symptoms or signs can predict an advanced CRC in the pre-operative stage.

**Methods:**

Retrospective analysis of 300 patients who underwent surgery for CRC between the years 2008 and 2019. Patients’ symptoms prior to CRC diagnosis were documented. Primary endpoint was the association of signs or/and symptoms with CRC diagnosis at TNM stages of 2–4 (i.e., highly advanced), compared to TNM score of 0–1 (i.e., locally advanced).

**Results:**

Three hundred patients, 91 with locally advanced and 209 with highly advanced CRC, were enrolled. There was a significant correlation between highly advanced CRC, compared to locally advanced, regarding tumor size (4.8 vs. 2.6 cm, *p*<0.001), presentation of any symptom prior to diagnosis (77% vs. 54%, *p*<0.001), anemia (46% vs. 29%, *p*=0.004), and severe anemia (17% vs. 4%, *p*=0.002). Mean hemoglobin was 12.2 ± 2.2 and 13.1 ± 1.8 in the highly advanced compared to locally advanced CRC, respectively, *p*<0.001. Anemia correlated with the T stage of the tumor: 21% of patients diagnosed at stages 0–1 had anemia, 39% at stage 2, 44% at stage 3, and 66% at stage 4 (*p*=0.001).

**Conclusions:**

Anemia is the only finding that correlates with highly advanced CRC, in the pre-operative stage. When CRC has been diagnosed, the presence of anemia, at any level, may be considered in determining prognosis at the pre-operative stage. Physicians should be aware that when anemia is present, the risk for highly advanced CRC increases, and therefore should pursue with CRC detection.

## Background

Colorectal cancer (CRC) is the third most commonly diagnosed cancer in the world among men (10.6% of the total cases) and the second among women (9.4%). It is also the second leading cause of cancer death worldwide (9.4%) [1]. The prognosis of CRC depends mainly on the stage of the tumor at the time of detection. For example, the 5-year relative survival of patients diagnosed with CRC ranges from 90%, for patients detected at a localized stage, to 11% for patients with distant tumor spread [2, 3]. CRC tumors progress slowly over years, from pre-cancerous adenomas to invasive cancer [4]. This makes CRC highly suitable for screening programs, which aim to trace and remove pre-cancerous adenomas or early-stage tumors. It has been established that, compared with symptom detected CRC, screening-detected CRC is associated with better prognosis [5, 6]. Unfortunately, despite the widespread use of public screening programs, in most cases, the disease is diagnosed only after the onset of symptoms [7, 8]. Moreover, many of the CRC-related symptoms, such as hematochezia, diarrhea, constipation, loss of weight, and abdominal pain, are also common with benign conditions, and almost half of cancer patients present with non-specific symptoms prior to diagnosis [8, 9]. Given that most CRCs initially present with non-emergent symptoms, it has been suggested to base the efforts of early diagnosis not only on screening, but also on improved recognition of symptomatic cancer [10]. Symptom-based screening may be helpful for the diagnosis of any CRC, but high risk compared to low-risk cancers are not specifically targeted. Previous studies have shown that hematochezia [9] and anemia [11, 12] are the presenting symptoms which have the highest positive predictive value (PPV) for detecting CRC, defining the stage of the tumor, and assessing the potential mortality. Hematochezia is commonly associated with less advanced staging [13, 14] and reduced mortality, while anemia is associated with more advanced staging and higher mortality rates [10]. Preoperative anemia was also significantly associated with decreased long-term overall survival and disease-free survival [15]. As opposed to other CRC symptoms, which could be vague, anemia is easier to define and follow. It is important to understand which symptoms or signs can predict highly advanced CRC. A physician needs to know when to insist on performing CRC screening and ensure compliance by the patient. We attempted to collectively assess all GI symptoms and signs related to CRC and prioritize them for risk of highly advanced CRC.

In this study, we checked which CRC-related symptoms or signs correlate with highly advanced CRC. These signs may assist the physician to allocate those patients that may need an urgent diagnosis and treatment. At the pre-operative stage, this may add in predicting the long-term prognosis.

## Methods

### Patients and study design

A retrospective study of 300 patients was conducted at the Shaare Zedek Medical Center (SZMC) in Jerusalem, Israel, between the years 2008 and 2019. Inclusion criteria included patients aged 50–75 years old who underwent surgery due to CRC. Exclusion criteria were patients who did not undergo surgery, either due to metastatic disease or other background diseases, as well as patients who underwent endoscopic excision of a tumor and did not require additional surgery. Patients were divided into two groups: Patients with a TNM score of 2–4 were considered highly advanced, and patients with TNM 0–1 were considered locally advanced. The study protocol was approved by the local Helsinki committee.

### Case definition

Colorectal cancer was defined as adenocarcinoma by a formal histopathological report. Each case had a complete TNM scoring. The final TNM was given only after surgery and completion of cross-sectional imaging. Tumor location was categorized into proximal colon (cecum and ascending colon), transverse colon (hepatic flexure to splenic flexure), distal colon (descending colon to sigmoid), and rectum. In addition, we collected data on the tumor size (defined by the largest dimension of the tumor found during surgery) and whether the tumor was an obstructing tumor.

We recorded which CRC-related symptoms appeared at the time of diagnosis, according to the medical records of the hospital, from the following list: melena, hematochezia, significant loss of weight, constipation, diarrhea, chronic abdominal pain, and severe abdominal pain. Abdominal pain was defined as severe if the patient was referred to the emergency room because of it. Significant loss of weight was defined as an unintentional loss of at least 5% of body weight within 6 months. Other symptoms were recorded according to the patients' reports. The hemoglobin level of each patient was documented from blood sample results taken the day before surgery. Anemia was defined as hemoglobin less than 13 g/dL in men, and hemoglobin less than 12 g/dL for women. Severe anemia was collectively defined as any hemoglobin level below 10 g/dL. Patients who were detected with CRC through the screening program only were considered asymptomatic.

### Statistical analysis

Descriptive statistics were used to compare baseline demographic characteristics of both groups. Patients’ data and clinical parameters are given as means with standard deviation (SD). For categorical variables, results are reported as absolute numbers with population proportions (percentages) in parenthesis or vice versa as indicated. To analyze differences in the distribution of categorical data, the chi-square test or Fisher exact test was used, as appropriate. Continuous variables were analyzed by the *t* test or the Mann-Whitney *U* test for normally or non-normally distributed variables, respectively. *P* values <0.05 were considered to indicate statistical significance. All statistical analyses were conducted using the SPSS 21.0 software (SPSS, Chicago, IL).

## Results

### Comparison between locally and highly advanced CRC characteristics

**Fig. 1:**
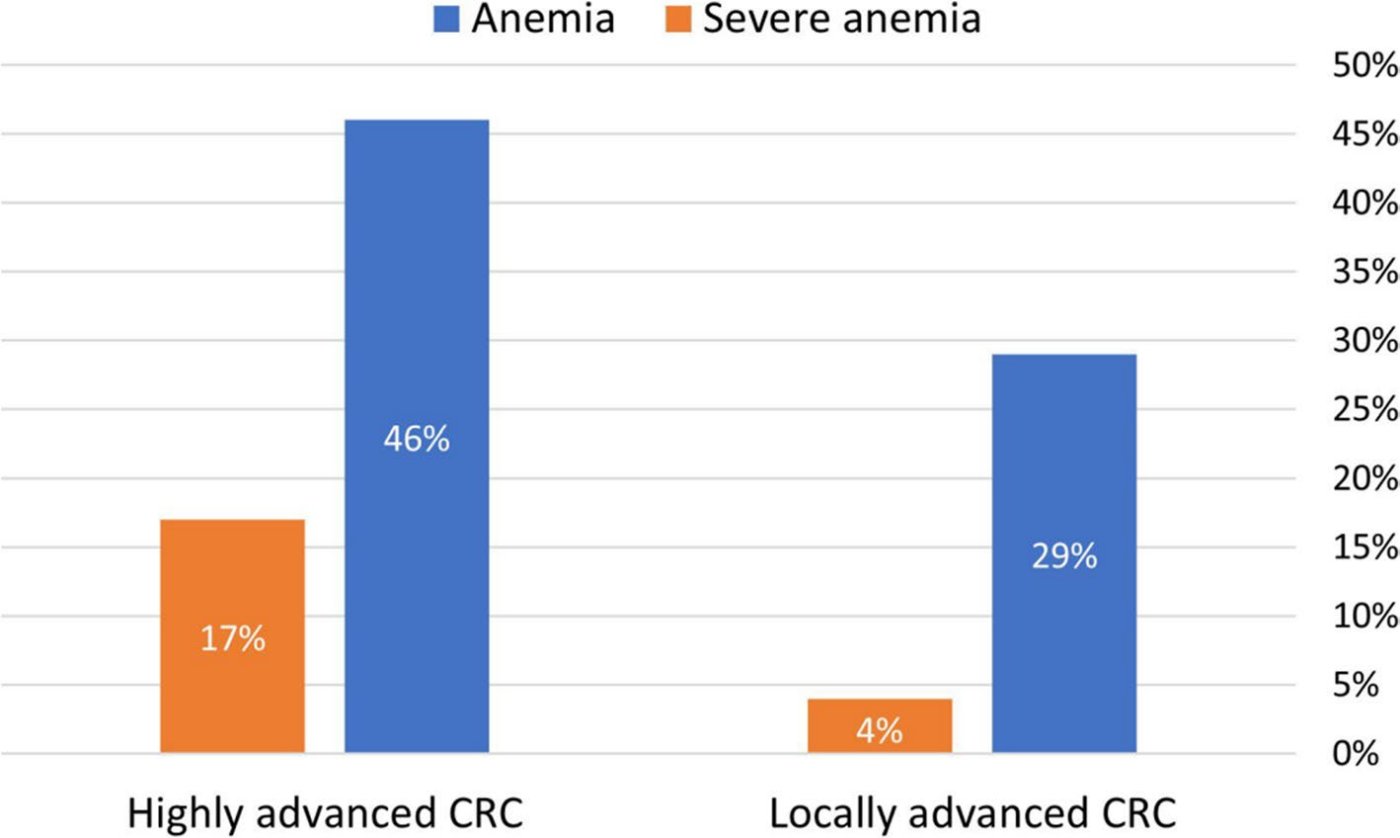
Percentage of anemia in the highly advanced CRC vs. locally advanced CRC groups. Both anemia and severe anemia, prior to surgery, were more common in the highly advanced CRC group compared to the locally advanced CRC group (46.4% vs. 28.6% and 17% vs. 4%, *p*=0.004 and *p*=0.002, respectively), demonstrating the correlation between preoperative anemia, at any level, and more advanced disease. CRC, colorectal cancer; ﻿Anemia—HgB < 13 g/dL for male or HgB < 12 g/dL for female; severe anemia—HgB < 10 g/dL﻿

Three hundred cases were studied, 91 locally advanced and 209 highly advanced CRC. Age and gender distributions were similar among both groups. In the highly advanced vs. locally advanced group, the tumors were more likely to be in the transverse and distal colon, larger, cause obstruction, and have an elevated CEA (Table 1). The highly advanced compared to locally advanced patients were less likely to be asymptomatic when first diagnosed (22.5% vs. 46%, *p* <0.001, respectively). Anemia and severe anemia prior to surgery were more common in highly advanced vs. locally advanced CRC (46.4% and 17% vs. 28.6% and 4%, *p*=0.004 and *p*=0.002, respectively) (Fig. 1).

**Table 1:** Clinicopathological characteristics of the study cohort, in patients with locally advanced and highly advanced CRC

Characteristics		Locally advanced CRC (N = 91)	Highly advanced CRC (N = 209)	***P*** value
Age (years), mean ± SD		63.7 ± 6.7	63.4 ± 7.1	0.684
Gender—female, *n* (%)		48 (53%)	91 (44%)	0.149
Tumor location	Proximal colon	20 (22%)	43 (21%)	0.002
	Transverse colon	11 (12%)	49 (23%)	
	Distal colon	31 (34%)	87 (42%)	
	Rectum	29 (32%)	28 (13%)	
	Multifocal	0	2 (1%)	
Tumor size (cm), mean ± SD		2.58 ± 1.59	4.83 ± 2.36	*p*<0.001
Obstructing tumor		1 (1.1%)	56 (27%)	*p*<0.001
Symptoms	Asymptomatic	42 (46%)	47 (22.5%)	*p*<0.001
	Symptomatic	49 (54%)	162 (77.5%)	
CEA > 5 (ng/ml), *n* (%)		5 (9%)	47 (34%)	0.0013
HgB (g/dL), mean ± SD		13.1 ± 1. 8	12.2 ± 2.2	*p*<0.001
Anemia, *n* (%)		26 (28.6%)	97 (46.4%)	0.004
Severe anemia, *n* (%)		4 (4%)	35 (17%)	0.0021

Anemia—HgB < 13 g/dL for male or HgB < 12 g/dL for female; severe anemia—HgB < 10 g/dL

*CRC* colorectal cancer, *HgB* hemoglobin, *CEA* carcinoembryonic antigen

### The correlation between CRC symptoms and advanced CRC

Two hundred eleven patients (70% of all CRC cases) suffered from disease-related symptoms prior to diagnosis. 76.8% of the patients who suffered from at least one symptom prior to diagnosis, were diagnosed with highly advanced CRC, while 23.8% of them had locally advanced CRC, *p*<0.001. When comparing each symptom separately, only severe abdominal pain and hematochezia were significantly different between the highly advanced and locally advanced groups (100% vs. 0% and 63.9% vs. 36.1%, *p*<0.001, respectively) (Table 2). All other symptoms were not associated with an increased risk for highly advanced CRC.

**Table 2:** Correlation between CRC symptoms and TNM staging

Symptoms	Number of cases (% of all symptomatic cases)	Locally advanced CRC, ***n*** (% within reported symptom)	Highly advanced CRC, ***n*** (% within reported symptom)	***P*** value
Any symptoms	211 (100%)	49 (23.2%)	162 (76.8%)	*p*<0.001
Melena	12 (5.7%)	3 (25%)	9 (75%)	0.881
Hematochezia	83 (39.3%)	30 (36.1%)	53 (63.9%)	*p*<0.001
Significant loss of weight	21 (10%)	3 (14.3%)	18 (85.7%)	0.418
Constipation	35 (16.6%)	5 (14.3%)	30 (85.7%)	0.170
Diarrhea	35 (16.6%)	8 (22.9%)	27 (77.1%)	0.955
Chronic abdominal pain	38 (18%)	6 (15.8%)	32 (84.2%)	0.231
Severe abdominal pain	27 (12.8%)	0 (0%)	27 (100%)	0.002

*CRC* colorectal cancer

### The association between preoperative anemia, TNM stage, and other clinicopathological characteristics

**Fig. 2:**
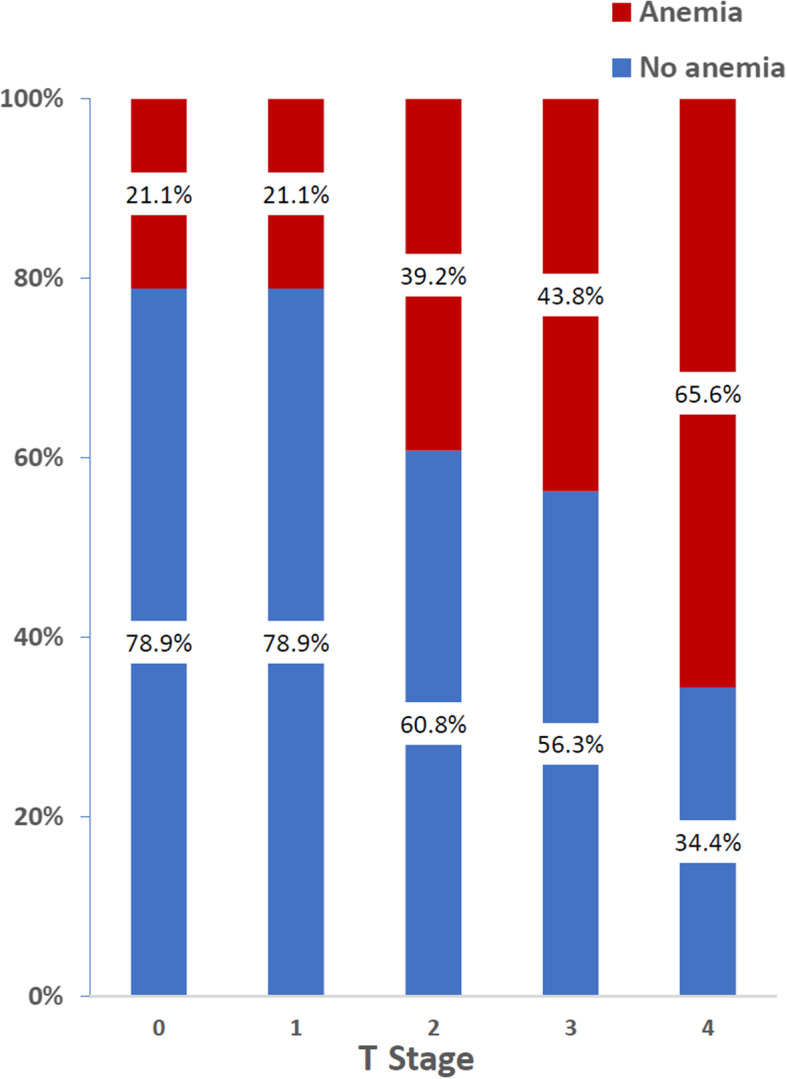
Percentage of preoperative anemia cases, according to T stage. Examination of the anemic patients' proportion, according to the T stage only, shows a prominent upward trend from T stage 0 to 4, with 21% anemic at stages 0–1, 39% at stage 2, 44% at stage 3, and 66% at stage 4 (*p*=0.001). Anemia—HgB < 13 g/dL for male or HgB < 12 g/dL for female

One hundred twenty-three patients (41%) had preoperative anemia. The age and sex distribution were not significantly different between the anemic and non-anemic patients. In the anemia group, more patients were diagnosed with highly advanced cancer (78.9% vs. 63.3%, *p*=0.004), proximal disease, larger and obstructive tumors (Table 3). When examining the percentage of anemic patients for each TNM score separately, we found that there is a prominent upward trend in TNM stages 0–2, that steadies and even turns downward in TNM stages 3–4: 22% of stage 0 cases were anemic, 30% of stage 1, 52% of stage 2, 42% of stage 3, and 44% of stage 4 (*p*=0.034). However, when examining T score only, in a similar way, the trend remains upward from T stage 1 to 4, with 21% anemic at stages 0–1, 39% at stage 2, 44% at stage 3, and 66% at stage 4 (*p*=0.001) (Fig. 2).

**Table 3:** Comparison between patients with and without preoperative anemia to CRC clinicopathological characteristics

Characteristics		No anemia (***N*** = 177)	Anemia (***N*** = 123)	***P*** value
Age (years), mean ± SD		63.15 ± 6.7	64.02 ± 7.3	0.291
Gender—female, *n* (%)		88 (49.7%)	51 (41.5%)	0.159
TNM stage, categorized	Locally advanced CRC	65 (36.7%)	26 (21.1%)	0.004
	Highly advanced CRC	112 (63.3%)	97 (78.9%)	
Tumor location	Proximal colon	28 (15.8%)	35 (28.4%)	0.016
	Transverse colon	32 (18.1%)	28 (22.8%)	
	Distal colon	81 (45.8%)	37 (30.1%)	
	Rectum	35 (19.8%)	22 (17.9%)	
	Multifocal	1 (0.5%)	1 (0.8%)	
Tumor size (cm) ± SD		3.59 ± 2.03	4.95 ± 2.64	*p*<0.001
Obstructing tumor, *n* (%)		25 (14.1%)	32 (56.1%)	0.010
Symptoms	Asymptomatic	69 (39%)	20 (16.3%)	*p*<0.001
	Symptomatic	108 (61%)	103 (83.7%)	

Anemia—HgB < 13 g/dL for male or HgB < 12 g/dL for female

*CRC* colorectal cancer

## Discussion

Previous studies have examined the relation between symptoms associated with CRC, tumor staging, and long-term prognosis. Studies that compared screening-detected CRC and symptom-related CRC, showed a significant survival advantage of the former group [16, 17], demonstrating the importance of early CRC diagnosis. We found that only 30% of the cases included in the study were asymptomatic and diagnosed through our CRC screening program. Symptoms associated with CRC tend to be vague and non-specific within the population, and therefore their diagnostic value is low. Out of the various symptoms examined in different studies, hematochezia [9] and anemia [11] stand out as the symptoms with the highest diagnostic value, regardless of age and other medications. Anemia was also shown to correlate with poor prognosis, but this was not compared to all other CRC-related symptoms. Unlike prior studies, our study did not examine symptoms as a predictive value for the presence of CRC, but rather for the severity of CRC. Similar to other studies [10], our results show that the prognostic value of most symptoms is not significant. When examining each of the symptoms separately, we discovered that only hematochezia and severe abdominal pain were statistically significant predicting factors of the TNM staging. Hematochezia stands out in previous studies [13, 14], and in this study as well, as a predictor of a better TNM stage. Yet, hematochezia is a somewhat problematic feature because it is associated with rectal cancer [13], in which neoadjuvant therapy is often given before surgery. Since the staging is defined only after surgery and is affected by the treatment given before the surgical treatment, it is not surprising that in many studies hematochezia is associated with a better TNM result. Thus, although hematochezia has a high value as a “red flag” in primary care which requires further inspection [12], its value as a symptom that accurately predicts prognosis is questionable and requires additional research. In addition, we found that all cases, in which a symptom of severe abdominal pain appeared, were associated with highly advanced CRC. These findings corroborate other studies [18, 19], which showed that emergent presentation of CRC was in correlation with more advanced histopathologic features, and therefore emphasize as well the significance of early diagnosis.

As opposed to most non-specific symptoms, anemia stands out as a laboratory finding, easy to measure and compare between patients worldwide, with a higher PPV of CRC relative to other symptoms [11]. Preoperative anemia is an effective and reliable tool for assessing prognosis via TNM staging, as it was also established in previous studies [15, 20, 21]. Our research shows that anemia as a whole, and specifically severe anemia is important for predicting highly advanced cancer. Anemia in CRC patients originates not only from occult or visible bleeding from the tumor itself, but also from a systemic inflammatory response [22]. We showed that there was a large difference in the proportion of anemic cases for TNM stages 0–2, while there was no significant difference when reaching TNM stages 3 and 4. Yet, when examining the T stage alone, the upward trend in the proportion of anemic cases remained throughout the ranking. This indicates that the presence of anemia is mainly due to the tumor penetration into nearby tissues and is less affected by the lymphatic and metastatic spread, which is defined as TNM stages 3 and 4. We found that whereas in patients presenting with anemia, where tumors tended to be located in the right colon, the tumors found in non-anemic patients were more likely to be located in the distal colon. Interestingly, unlike other studies [20, 23], in our study, the percent of rectal tumor cases was similar in both the anemic and the non-anemic groups. In addition, we found that anemia cases were associated with larger tumor size and a higher chance of an obstructing tumor, both of which are also associated with worse prognosis [24, 25].

In conclusion, we found that patients with highly advanced CRC are different from patients with locally advanced CRC not only in several parameters of tumor location and size, but also in characteristics which can be identified before surgery. Thus, patients with more advanced cancer tend to be more symptomatic before diagnosis, and with a higher percentage of anemia. In contrast to most of the clinically manifested symptoms, we found a consistent association between the preoperative hemoglobin level and the TNM score. The higher the TNM stage, the higher the likelihood of preoperative anemia. This trend was even more pronounced when examining the T stage separately. We have shown that anemia, as opposed to all other symptoms, was the only finding to clearly correlate with highly advanced CRC. The primary care physician should be aware that when anemia is present, the risk for highly advanced CRC increases, and therefore should insist to pursue with CRC detection. When CRC has been diagnosed, the presence of anemia, of any level, may be considered determining prognosis at the pre-operative stage.

Several limitations should be considered regarding the current study. First, it was conducted on a relatively small sample of 300 patients, all of them from a single medical center; thus, the impact of the results on diverse populations should be considered cautiously. Second, the data on patients' symptoms was retrospectively taken from patients' reports, as documented in the hospital medical reports. It is not unlikely that some of the symptoms may have been not adequately reported either by the patients themselves or by the physicians. Also, since the documentation was performed at the time of admission, which in most of the cases occurred following diagnosis of CRC, it is possible that the medical staff did not see paramount importance in documenting the symptoms accurately, including symptom duration. Yet, the main findings of the study are based on well-documented numerical data, such as blood test results in terms of anemia, and tumor staging and characteristics which originated from reliable histopathological reports and CT scans.

In summary, any level of anemia, but no other GI symptom, correlates with highly advanced CRC and poor prognosis. The physician should acknowledge this for prioritizing patients for CRC screening. Anemia should also be considered as an integrated part of the pre-operative staging assessment for CRC.

## Data Availability

The datasets used and/or analyzed during the current study are available from the corresponding author on reasonable request.

## Abbreviations

CRC: Colorectal cancer
PPV: Positive predictive value
CEA: Carcinoembryonic antigen
CT: Computed tomography
TNM: Tumor-Nodes-Metastasis
GI: Gastrointestinal

## References

[1] Sung H, Ferlay J, Siegel RL, Laversanne M, Soerjomataram I, Jemal A (2021). Global Cancer Statistics 2020: GLOBOCAN Estimates of Incidence and Mortality Worldwide for 36 Cancers in 185 Countries. CA Cancer J Clin.

[2] Brenner H, Kloor M, Pox CP (2014). Colorectal cancer. Lancet..

[3] Miller KD, Siegel RL, Lin CC, Mariotto AB, Kramer JL, Rowland JH (2016). Cancer treatment and survivorship statistics, 2016. CA Cancer J Clin.

[4] Wu Z, Li Y, Zhang Y, Hu H, Wu T, Liu S (2020). Colorectal Cancer Screening Methods and Molecular Markers for Early Detection. Technol Cancer Res Treat.

[5] Amri R, Bordeianou LG, Sylla P, Berger DL (2013). Impact of screening colonoscopy on outcomes in colon cancer surgery. JAMA Surg.

[6] Leijssen LGJ, Dinaux AM, Kunitake H, Bordeianou LG, Berger DL. Detrimental impact of symptom-detected colorectal cancer. Surg Endosc. 2019;(0123456789). 10.1007/s00464-019-06798-8. 10.1007/s00464-019-06798-8 31020436

[7] Weller D, Menon U, Zalounina Falborg A, Jensen H, Barisic A, Knudsen AK, et al. Diagnostic routes and time intervals for patients with colorectal cancer in 10 international jurisdictions; Findings from a cross-sectional study from the International Cancer Benchmarking Partnership (ICBP). BMJ Open. 2018;8(11):1–18. 10.1136/bmjopen-2018-023870 PMC6278806 30482749

[8] Juul JS, Hornung N, Andersen B, Laurberg S, Olesen F, Vedsted P. The value of using the faecal immunochemical test in general practice on patients presenting with non-alarm symptoms of colorectal cancer. Br J Cancer. 2018;119(4). 10.1038/s41416-018-0178-7. 10.1038/s41416-018-0178-7 PMC6133998 30065255

[9] Rasmussen S, Haastrup PF, Balasubramaniam K, Elnegaard S, de Christensen RP, Storsveen MM (2019). Predictive values of colorectal cancer alarm symptoms in the general population: a nationwide cohort study. Br J Cancer.

[10] Stapley S, Peters TJ, Sharp D, Hamilton W (2006). The mortality of colorectal cancer in relation to the initial symptom at presentation to primary care and to the duration of symptoms: a cohort study using medical records. Br J Cancer.

[11] Astin M, Griffin T, Neal RD, Rose P, Hamilton W (2011). The diagnostic value of symptoms for colorectal cancer in primary care: a systematic review. Br J Gen Pract.

[12] Ewing M, Naredi P, Zhang C, Månsson J (2016). Identification of patients with non-metastatic colorectal cancer in primary care: a case-control study. Br J Gen Pract.

[13] Korsgaard M, Pedersen L, Sørensen HT, Laurberg S (2006). Reported symptoms, diagnostic delay and stage of colorectal cancer: a population-based study in Denmark. Color Dis.

[14] Alexiusdottir KK, Möller PH, Snaebjornsson P, Jonasson L, Olafsdottir EJ, Björnsson ES (2012). Association of symptoms of colon cancer patients with tumor location and TNM tumor stage. Scand J Gastroenterol.

[15] Wilson MJ, van Haaren M, Harlaar JJ, Park HC, Bonjer HJ, Jeekel J (2017). Long-term prognostic value of preoperative anemia in patients with colorectal cancer: a systematic review and meta-analysis. Surg Oncol.

[16] Kubisch CH, Crispin A, Mansmann U, Göke B, Kolligs FT (2016). Screening for colorectal cancer is associated with lower disease stage: a population-based study. Clin Gastroenterol Hepatol.

[17] Brenner H, Jansen L, Ulrich A, Chang-Claude J, Hoffmeister M (2016). Survival of patients with symptom- and screening-detected colorectal cancer. Oncotarget..

[18] Amri R, Bordeianou LG, Sylla P, Berger DL (2015). Colon cancer surgery following emergency presentation: effects on admission and stage-adjusted outcomes. Am J Surg.

[19] Ghazi S, Berg E, Lindblom A, Lindforss U (2013). Clinicopathological analysis of colorectal cancer: a comparison between emergency and elective surgical cases. World J Surg Oncol.

[20] Mörner MEM, Edgren G, Martling A, Gunnarsson U, Egenvall M (2017). Preoperative anaemia and perioperative red blood cell transfusion as prognostic factors for recurrence and mortality in colorectal cancer—a Swedish cohort study. Int J Color Dis.

[21] Tokunaga R, Nakagawa S, Miyamoto Y, Ohuchi M, Izumi D, Kosumi K (2019). The impact of preoperative anaemia and anaemic subtype on patient outcome in colorectal cancer. Color Dis.

[22] Väyrynen JP, Tuomisto A, Väyrynen SA, Klintrup K, Karhu T, Mäkelä J (2018). Preoperative anemia in colorectal cancer: Relationships with tumor characteristics, systemic inflammation, and survival. Sci Rep.

[23] McSorley ST, Johnstone M, Steele CW, Roxburgh CSD, Horgan PG, McMillan DC (2019). Normocytic anaemia is associated with systemic inflammation and poorer survival in patients with colorectal cancer treated with curative intent. Int J Color Dis.

[24] Atsushi I, Mitsuyoshi O, Kazuya Y, Syuhei K, Noriyuki K, Masashi M (2016). Long-term outcomes and prognostic factors of patients with obstructive colorectal cancer: a multicenter retrospective cohort study. World J Gastroenterol.

[25] Dai W, Li Y, Meng X, Cai S, Li Q, Cai G (2017). Does tumor size have its prognostic role in colorectal cancer? Re-evaluating its value in colorectal adenocarcinoma with different macroscopic growth pattern. Int J Surg.

